# KADAIF: an anomaly detection method for complex microbiome data

**DOI:** 10.1093/bioinformatics/btaf520

**Published:** 2025-09-19

**Authors:** Omri Peleg, Maya Raytan, Elhanan Borenstein

**Affiliations:** Blavatnik School of Computer Science and AI, Tel Aviv University, Tel Aviv, 6997801, Israel; Blavatnik School of Computer Science and AI, Tel Aviv University, Tel Aviv, 6997801, Israel; Blavatnik School of Computer Science and AI, Tel Aviv University, Tel Aviv, 6997801, Israel; Gray Faculty of Medical & Health Sciences, Tel Aviv University, Tel Aviv, 6997801, Israel; Santa Fe Institute, Santa Fe, NM, 87501, United States

## Abstract

**Motivation:**

The gut microbiome plays an important role in human health and disease, prompting large-scale studies that generate extensive datasets. A critical preprocessing step in analysing such datasets is anomaly detection, which aims to identify erroneous samples and prevent misleading statistical outcomes. Microbiome data, however, pose unique challenges such as compositionality, sparsity, interdependencies, and high dimensionality, limiting the effectiveness of conventional methods and highlighting the need for specifically-tailored approaches for anomaly detection in microbiome data.

**Implementation:**

To address this challenge, we introduce KADAIF, a microbiome-specific anomaly detection method that generalizes the common Isolation Forest (IF) approach. As in IF, KADAIF builds an ensemble of trees, each recursively partitioning the data along randomly selected features, and measures the average depth at which samples are isolated, assuming that anomalous samples will be isolated closer to the root. Unlike IF, however, KADAIF partitions samples based on subsets of features (coupled with dimensionality reduction), addressing microbiome-specific properties such as sparsity and species interactions.

**Results:**

We evaluate KADAIF by simulating common scenarios that introduce anomalous behavior, demonstrating that KADAIF outperforms alternative methods across various settings and datasets. Furthermore, we show that KADAIF outperforms IF in detecting anomalies also in other types of high-dimensional sparse biological data. Finally, we show KADAIF’s application for identifying disease onset in longitudinal microbiome data and for partitioning cases versus controls based on the Anna Karenina principle. Combined, our work highlights KADAIF's potential to enhance microbiome data processing and downstream analyses, with beneficial implications for precision medicine studies.

**Availability and implementation:**

An implementation of KADAIF, as well as all the code used for the analysis, is available on GitHub (https://github.com/borenstein-lab/KADAIF).

## 1 Introduction

The human microbiome, and in particular the human gut microbiome, the ensemble of microorganisms that inhabit the human gut, plays a well-established role in various health conditions (Gilbert *et al.* 2018, Fan and Pedersen 2021, Shanahan *et al.* 2021). This role of the gut microbiome in our health has prompted numerous extensive studies of this complex system, profiling the composition of the microbiome across diverse cohorts and disease states (Kostic *et al.* 2014, Shreiner *et al.* 2015, Jiang *et al.* 2017, Wandro *et al.* 2018, Yachida *et al.* 2019). Such studies, in turn, have generated numerous large-scale datasets that can be processed and analysed to obtain novel insights about the microbiome in health and disease (Huttenhower *et al.* 2012, Methé *et al.* 2012, Zhernakova *et al.* 2016, McDonald *et al.* 2018, Proctor *et al.* 2019, Fromentin *et al.* 2022).

One related task, which is a common practice in the preprocessing phase of data obtained from large-scale clinical studies, is *anomaly detection* (also known in various domains as *outlier detection*) (Fitriyani *et al.* 2019, Ripan *et al.* 2021, Shaikh *et al.* 2023). Anomalies refer to data samples that deviate in their characteristics from the majority of samples in the dataset in some manner, potentially indicating various issues such as measurement errors, mislabeling, contamination, or batch effects. These anomalies, if ignored, can introduce false signals or obscure true biological patterns, ultimately giving rise to misleading or confusing outcomes. Detecting such abnormal samples and accounting for them in downstream analyses, e.g. by removing them from the dataset, correcting anomalous value, reducing their weights in predictive models, or applying analytical approaches that are more robust to outliers, is therefore crucial.

With that in mind, a variety of anomaly detection algorithms have been previously introduced. Such algorithms typically apply unsupervised or semi-supervised approaches and can be generally categorized into several distinct groups (Chandola *et al.* 2009). These include: (i) classification-based techniques, which leverage classification models to identify anomalies; (ii) nearest neighbors-based techniques, which examine the proximity of samples to identify outliers; (iii) clustering-based techniques, which group similar samples and detect deviations from identified clusters; (iv) statistical techniques, which model data behavior to detect anomalous data points; and (v) spectral techniques, which utilize dimensionality reduction to identify irregular samples.

In the microbiome domain, many studies unfortunately completely overlook the task of detecting anomalies prior to conducting downstream analysis, partly due to the lack of a well-established and standard protocol for anomaly detection in microbiome data. Amongst the relatively few microbiome studies that do attempt to explicitly incorporate anomaly detection (Bellocchi *et al.* 2019, Leigh *et al.* 2021, Liñares-Blanco *et al.* 2022, Banjac *et al.* 2023), most apply general-purpose methods, with perhaps the most commonly employed approach being *Isolation Forest (IF)* (Liu *et al.* 2008). This popular unsupervised non-parametric anomaly detection method is based on the construction of multiple random *isolation trees*, each recursively partitioning the data space using random splitting points along randomly selected features. Anomalies, which are generally assumed to be unique, rare, and with atypical feature values, are expected to be isolated along these trees with fewer partitions (and thus closer to the root of the tree) compared to normal samples. The average depth (i.e. the distance from the root) at which a sample was isolated in this forest of random isolation trees can thus serve as an anomaly score, with lower average depth suggesting anomalous samples. Several generalizations of this concept have been also introduced (Sun *et al.* 2019, Li *et al.* 2020, Chabchoub *et al.* 2022), including, for example, Extended Isolation Forest (Hariri *et al.* 2021) which uses a random slope and intercept at each branching node, thus enabling non-axis-parallel splits and a more flexible and nuanced sample partitioning.

Importantly, however, microbiome data are characterized by several unique properties, including specifically compositionality (Gloor *et al.* 2017), sparsity (Tsilimigras & Fodor 2016, Pan 2021), and high dimensionality (Li 2015, Tsilimigras and Fodor 2016), and accordingly often require specially tailored analytical methods for their curation, processing, and analysis. These unique characteristics of microbiome data are particularly challenging for anomaly detection and likely account for the limited use of such methods in this domain. For example, in high-dimensional and sparse spaces, distances and neighborhoods become less informative (Beyer *et al.* 1999), hindering the effectiveness of traditional neighbors-based or clustering-based techniques for detecting anomalies. Similarly, since microbiome data are difficult to model statistically (Aldirawi *et al.* 2023, Hajihosseini *et al.* 2025), general-purpose statistical techniques may fail to accurately capture the structure and interdependencies in the data, leading to ineffective or erroneous anomaly detection. IF is also poorly suitable, despite its popularity, for detecting anomalies in sparse, high-dimensional, and noisy data (Liao and Luo 2019). Specifically, IF only considers a single feature in each partition, and therefore lacks the ability to capture complex data patterns or dependencies between different features in the microbiome (Chabchoub *et al.* 2022, Xu *et al.* 2023), a likely scenario when shifts occur at the community level (Davenport *et al.* 2014).

To address this gap, a handful of microbiome-specific anomaly detection algorithms have been developed to date. One example is CLOUD (Montassier *et al.* 2018), an anomaly detection test that employs a non-parametric nearest neighbors approach and determines whether a microbiome sample is normal by measuring its proximity to a small number of normal reference samples. This method was used in several microbiome studies for dysbiosis detection (Saffouri *et al.* 2019, Wei *et al.* 2022) and for anomaly detection (Palmieri *et al.* 2022, Leng *et al.* 2024), yet, it assumes that the reference population used for comparison does not itself include any anomalies, which is a challenging requirement and likely hard to guarantee. Furthermore, CLOUD’s results markedly depend on the specific distance metric used, with many such metrics being uninformative in high-dimensional settings, as mentioned above. Other efforts in this field include AMAnD, a neural network framework designed to detect anomalies in metagenomic data (Price and Russell 2023), and a Bayesian modeling framework, suggested by Josephs *et al.* (2023). A distinct, yet related research area focuses on developing methods for detecting “novelty” in microbiome samples (Su *et al.* 2018, 2020), a task that aims to identify previously unobserved patterns in the data. For example, Su *et al.* (2018) developed a microbiome novelty score (MNS) to assess the similarity of microbiome samples to previously sequenced ones. They later (Su *et al.* 2020) utilized this score to identify and classify samples from individuals with different diseases. While potentially also applicable for anomaly detection, such novelty analyses again depend on large, pre-existing microbiome datasets with similar properties to the data being analysed, an assumption that is rarely met.

**Figure 1.**
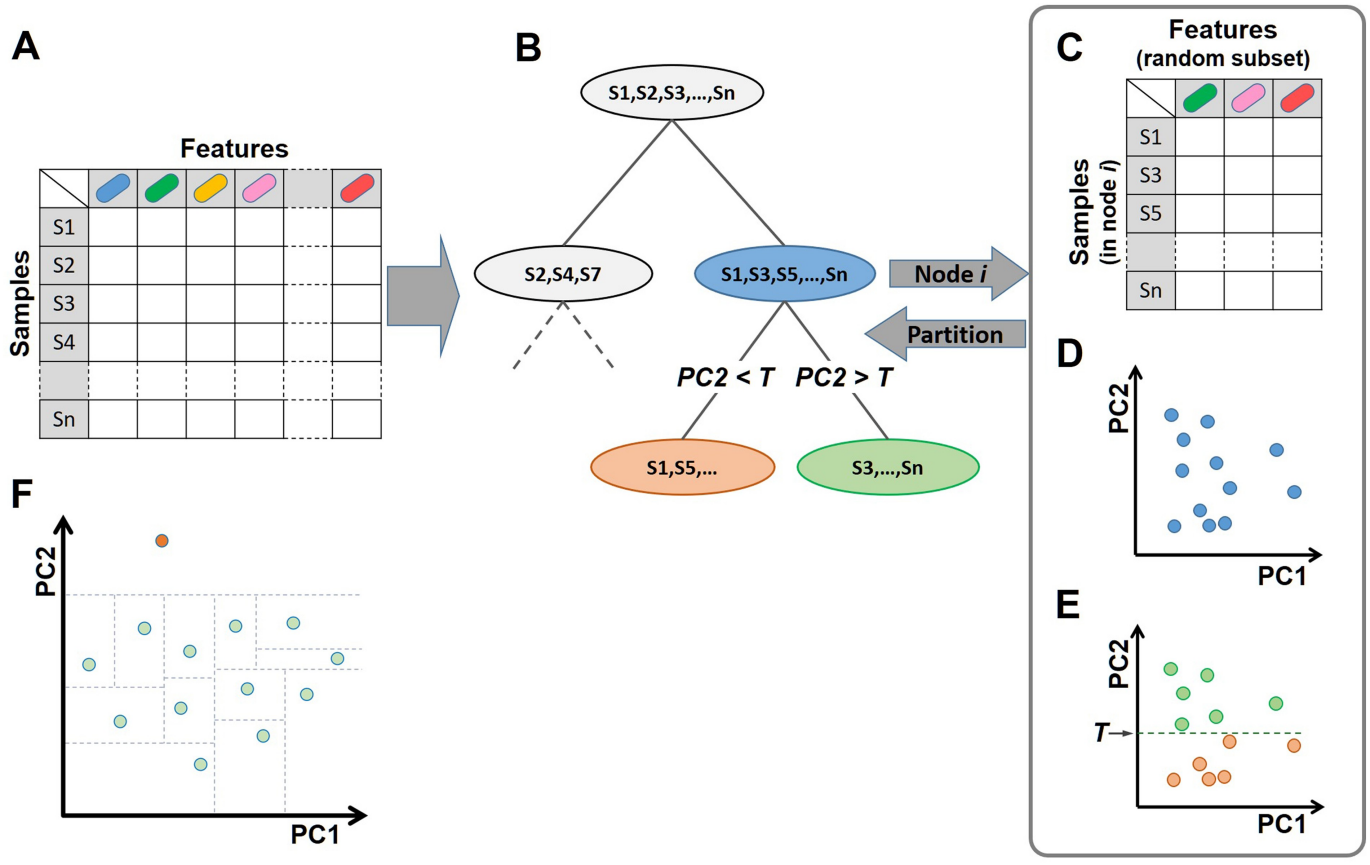
Schematic illustration of KADAIF. (A) KADAIF gets a feature table as input, which, in the case of microbiome data, lists the abundance of each taxon in each sample. (B) KADAIF then starts building a forest of random isolation trees, where each node partitions samples into two children nodes according to certain features. Specifically, KADAIF first selects a random subset of features (C), applies a dimensionality reduction method (e.g. PCoA) to this subset (D), and uses the projection on some randomly selected component to partition samples with a randomly selected threshold *T* (E). (F) KADAIF, like IF, operates under the assumption that anomalies are unique, rare, and distinct, and thus tend to be isolated with fewer random splits (illustrated by dashed lines) in the isolation tree. In this illustrative example, the topmost sample represents an anomaly and can indeed be easily isolate by a single random split (note also the large gap along PC2 between this sample and the rest, which allows for a wide range of random thresholds to isolate it). In contrast, most normal samples require multiple, highly specific splits to achieve full isolation at random.

Here, we aim to address these caveats and present a new microbiome-specific unsupervised anomaly detection method. Our method, which we term KADAIF (*K*-dimensional Anomaly Detection using Aggregated Isolation of Features), generalizes IF by selecting *multiple* random features and using dimensionality reduction for the splitting process (Fig. 1). We evaluate KADAIF by simulating data with mislabeling or contamination, two common scenarios that cause anomalous behavior, and show that KADAIF outperforms IF and CLOUD across multiple datasets. Furthermore, we show that KADAIF outperforms IF also when applied to other types of high-dimensional and sparse biological data, that it can be further used for identifying disease onset or abrupt shifts in longitudinal microbiome studies, and that it partitions cases versus controls based on increased variability in diseases.

## 2 Materials and Methods

### 2.1 The KADAIF algorithm

KADAIF is a tree-based ensemble anomaly detection algorithm that, similarly to the standard IF technique, identifies anomalies by calculating the average depth at which each sample is isolated in a forest of t random trees. However, in contrast to the standard algorithm, during the recursive tree building process, instead of selecting one feature at random in each node to partition samples, KADAIF randomly selects a *subset* of ψ features, renormalizes this feature subset (to get a proper relative abundance profile), applies some dimensionality reduction method to this subset (specifically, Principal Coordinates Analysis, PCoA, based on the Bray–Curtis distance matrix in the case of microbiome data), and uses the projection on the first component obtained, C (but see alternative approaches below), to partition the samples in that node with a randomly selected threshold. This process is repeated iteratively, until a node contains r samples at most (in our case r=1), or when the resulting tree is of maximum depth l (in our case l=#samples) (Fig. 1 and Box 1). Importantly, these subsampling and dimensionality reduction steps allow KADAIF to capture complex and potentially intricate relationships between features, thereby taking into account intrinsic dependencies in the data that may be overlooked by standard IF that partitions based on a single feature. Specifically, this strategy reduces reliance on any single, potentially sparse, noisy, or uninformative taxon, thereby mitigating the impact of sparsity in high-dimensional microbiome data. Moreover, by evaluating different combinations of taxa across splits, KADAIF can implicitly capture (albeit randomly) dependencies between features and community-level interactions. This is key to identifying anomalies that are manifested not by an aberrant shift in the distribution of a specific microbial taxon, but rather from coordinated community-wide changes in the abundances of multiple taxa or in the way they correlate with one another. Similarly, by using an ecological distance metric, like Bray–Curtis, to compute distances between *subsets* of taxa, KADAIF implicitly measures the similarity of internal proportions between samples, where each taxon is always considered within the context of others, thus inherently incorporating compositionality constraints into its anomaly detection process.

As in the standard IF paradigm, the expected rarity and distinctiveness of anomalies suggest that they should be easier to randomly isolate (see Fig. 1F), and are consequently expected to be isolated, on average, at shallower depths in the constructed trees compared to normal samples. We thus follow the approach outlined in the original IF method (Liu *et al.* 2008), and assign each sample x an anomaly score 0≤s(x)≤1, based on the average depth in the forest at which x was isolated, E(d(x)), and the average path length of an “unsuccessful search” in a binary search tree that with n input samples, c(n). Specifically, s(x) is calculated as follows (Preiss 1999):


$$s(x)=2^{\frac{E(d(x))}{c(n)}},\;c(n)=2(H(n-1)-\frac{n-1}{n})$$

where H is the harmonic number. Therefore, c(n) represents the average number of random splits required to isolate an arbitrary sample in a dataset of size n, while s(x) quantifies how easily sample x is isolated relative to this random baseline. Following this normalization, the closer s(x) is to 1, the probability that x is an anomaly is higher.

Box 1: Pseudo code of KADAIF anomaly detection algorithm.

KADAIF(X, t, l, ψ, r)

**Inputs:**  X-data, t-number of trees, l-maximal depth, ψ-subsampling size,r-minimal number of samples in a node

**Output:** A forest of KADAIF trees

1: Initialize Forest

2: For i = 1 to t do

3:    Forest ← Forest ∪MicrobiomeIsolationTree(X,0,l, ψ,r)

4: End for

 MicrobiomeIsolationTree (X, d, l, ψ, r)

**Inputs:**  X-data, d- current depth, l-maximal depth, ψ-subsampling size,r-minimal number of samples in a node

**Output:**  tree

1: **if**  d≥l or |X|<r then

2:    stop

3: **else** 

4:       features ← sample(X,ψ)

5:       features ←renormalize(features)

6:    C←projection_on_dimensionality_reduction_first_component(features)

7:    T←draw from U(min⁡{C},max⁡{C})

8: $\;\;\;X_{l}\leftarrow \mathrm{filter}(X[P\leq T])$

9: $\;\;\;X_{r}\leftarrow \mathrm{filter}(X[P>T])$

10: $\mathrm{Left}\;\leftarrow \mathrm{MicrobiomeIsolationTree}\;(X_{l},\;d+1,\;l,\;\psi ,\;r)$

11: $\mathrm{Right}\leftarrow \mathrm{MicrobiomeIsolationTree}\;(X_{r},\;d+1,\;l,\;\psi ,\;r)$

12: **end if** 

Since the KADAIF algorithm involves several parameters and can be configured in various ways, we first sought to examine which configuration yields the best performance. To this end, we have tested how well KADAIF separates anomalies from normal samples in a simulated mislabeling scenario (as defined below). The parameters assessed included the number of features that are subsampled in each node during the tree construction process, whether all features are drawn uniformly or based on their relative mean abundance in the dataset, whether the subsampling process is done with or without replacement, and whether data partitioning is done based on just one PC or on multiple PCs (potentially capturing regularities that do not appear in the first PC). To assess the latter parameter, we compared the naïve version of KADAIF that is based on the first PC alone, to versions where one of the first 20 PCs is used, with two alternative settings. In the first setting, one of these 20 PCs is drawn *with a uniform distribution* at each node, and in the second setting, one of these 20 PCs is drawn with a probability proportional to the variance explained by each PC (or, in the case of NMDS, to the relative decrease in stress of each PC—the equivalent of variance explained). Following a comprehensive analysis (see Note 1, available as supplementary data at *Bioinformatics* online), we opted to draw ∼1% of the features in the dataset in each node (with a minimum of 10 features), uniformly, and with replacement, and to randomly choose one PC from the first 20 PCs with probability proportional to the variance explained by each PC. Further evaluating the impact of different dimensionality reduction methods and distance metrics for the splitting process (see Note 2, available as supplementary data at *Bioinformatics* online), we similarly opted to use KADAIF’s default strategy noted above, relying on PCoA with Bray–Curtis distances.

### 2.2 Simulating normal and anomalous samples

Anomalies in a microbiome dataset may reflect various scenarios, including, most notably, mislabeling or contamination. Importantly, however, datasets with confidently labeled anomalies that could be used as ground truth for evaluating KADAIF’s ability to detect anomalies in such settings are challenging to obtain. We thus used a series of simulation-based analyses, aiming to assess the performances of KADAIF and of other anomaly detection methods, in identifying mislabeled or contaminated samples.

To this end, we used several publicly available microbiome datasets with diverse properties. The first dataset was obtained from Wang *et al.* (2020), and includes 287 shotgun-based samples, all obtained from different individuals, 220 of whom with an end-stage renal disease (ESRD). The second dataset was obtained from Franzosa *et al.* (2019), and includes 220 shotgun-based samples, also from different individuals, of whom 164 diagnosed with inflammatory bowel disease (IBD). In our analysis of these two case-control datasets, we utilized genus-level data, processed and curated as part of a dataset collection we recently published (Muller *et al.* 2022). We have also obtained data from Caporaso *et al.* (2011), a longitudinal 16S-based study that tracked the microbiome composition of two individuals (referred to as M3 and F4) over periods of 15 and 6 months, respectively, with nearly daily sampling (a total of 332 samples for M3 and 130 for M4). Our analysis relied on genus-level data processed by the original study. Finally, we used data from the Human Microbiome Project (HMP) (Huttenhower *et al.* 2012), including 16S-based samples obtained from various body sites of healthy individuals, including gut (418 samples), as well as various other body sites (5310 samples in total), to comprehensively characterize microbial communities associated with different regions. This dataset was processed using QIIME2 (Bolyen *et al.* 2019). Combined, these datasets include both 16S and shotgun-based sequencing data, collected from both case-control and longitudinal studies, underscoring KADAIF’s applicability across a broad range of settings and study designs.

To simulate anomalies, in each of the above studies we designated one group of samples as normal and the other as anomalous. Specifically, in case-control datasets, samples from healthy individuals were classified as normal and those from individuals with a disease were classified as anomalous. In the longitudinal dataset, samples from one of the two individuals were classified as normal and those from the other as anomalous (and *vice versa* in a second simulation). In the HMP dataset, gut samples were classified as normal, and samples from other body sites were classified as anomalies. Given these classifications, we simulated mislabeling scenarios (by including a few samples from the anomalous group in a larger set of normal samples) and contamination scenarios (by “contaminating” a few normal samples with traces of anomalous samples) as described below.

## 3 Results

### 3.1 Evaluating KADAIF’s ability to detect mislabeling-based anomalies

To simulate mislabeling scenarios, we constructed a pool of 50 samples from each dataset, wherein the majority of these 50 samples are obtained at random from the group labeled as normal, and a few samples are obtained from the group labeled as anomalous, which aligns with the inherent and expected rarity of anomalies in real-world datasets. This setup reflects a scenario in which a few samples out of a large set of samples are in fact mislabeled or swapped, thus introducing a few atypical samples (e.g. from individuals with a certain disease) into an otherwise homogenous dataset with predominantly normal (e.g. healthy) samples. The objective in this simulation is therefore to assess whether the various anomaly detection methods can identify these mislabeled anomalous samples based on their deviation from the majority group, which aligns with the inherent and expected rarity of anomalies in real-world datasets. We thus applied an anomaly detection algorithm to this pool, assigning each sample an anomaly score. This process was repeated 50 times, resulting in 2500 anomaly scores for each dataset and for each anomaly detection algorithm, allowing us to assess the ability of each algorithm to differentiate between normal and anomalous samples. The performance of each algorithm was then quantified by the area under the curve (AUC) of the receiver operating characteristic (ROC) curve based on these 2500 samples.

**Figure 2.**
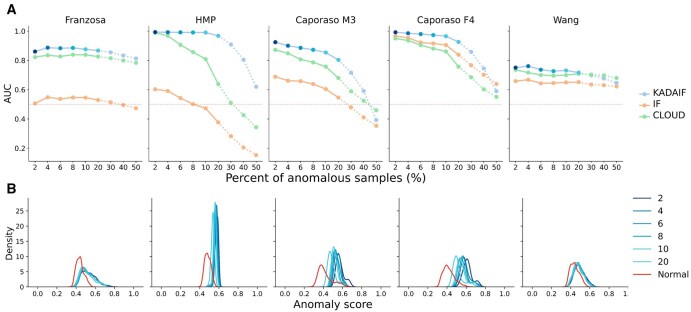
KADAIF’s performance across different anomaly percentages and datasets. (A) Comparison between the performances of KADAIF, IF, and CLOUD across different datasets and anomaly percentages. (B) Distributions of KADAIF’s anomaly scores for samples labeled as normal and anomalous across datasets and anomaly percentages. The distribution of anomaly scores for normal samples is illustrated only for the case of 2% anomalies (and see Note 1, available as supplementary data at *Bioinformatics* online). Distributions of anomaly scores for very high anomaly percentages (>20%) were omitted for clarity.

We specifically compared the performances of KADAIF to those of standard IF, which is both the most commonly used method and the basis of our algorithm, and of CLOUD, which is the main microbiome-specific anomaly detection algorithm introduced to date. To further examine how the rarity of anomalies impacts our ability to detect them, we simulated pools with varying percentage of anomalous samples, ranging from 2% to 50% (and see Section 3). Our results (Fig. 2A) suggest that KADAIF outperforms IF and CLOUD across all datasets and almost all anomaly percentage tested. Notably, in most cases, KADAIF exhibits substantially improved performance compared to IF, achieving AUC values exceeding 0.9, especially when the percentage of anomalous samples is low. A particularly intriguing observation from this analysis is evident in the Franzosa *et al.* dataset, where even when the percentage of anomalous samples reaches 50%, KADAIF's (as well as CLOUD’s) ability to detect these samples remains relatively high, with an AUC of 0.82. This phenomenon might be a manifestation of the Anna Karenina principle (Zaneveld *et al.* 2017, Ma 2020) where normal samples, taken from healthy individuals tend to be similar to each other, while those obtained from individuals with a given disease tend to exhibit greater variation.

Next, we sought to identify an anomaly score threshold that could be used to effectively filter out the majority of anomalous samples while dropping only a minimal number of normal samples. Such a threshold could be applied, e.g. when the proportion of anomalous samples is unknown. To this end, we have examined the distribution of anomaly scores obtained for anomalous and normal samples across these datasets and configurations (Fig. 2B). Not surprisingly, anomaly scores demonstrate a tendency to decrease with an increase in anomaly percentage, while the distribution of anomaly scores for normal samples remains relatively stable (Note 1, available as supplementary data at *Bioinformatics* online). Based on these distributions, we propose that samples with anomaly scores >0.5 should be flagged as potential anomalies.

### 3.2 Evaluating KADAIF’s ability to detect contamination

**Figure 3.**
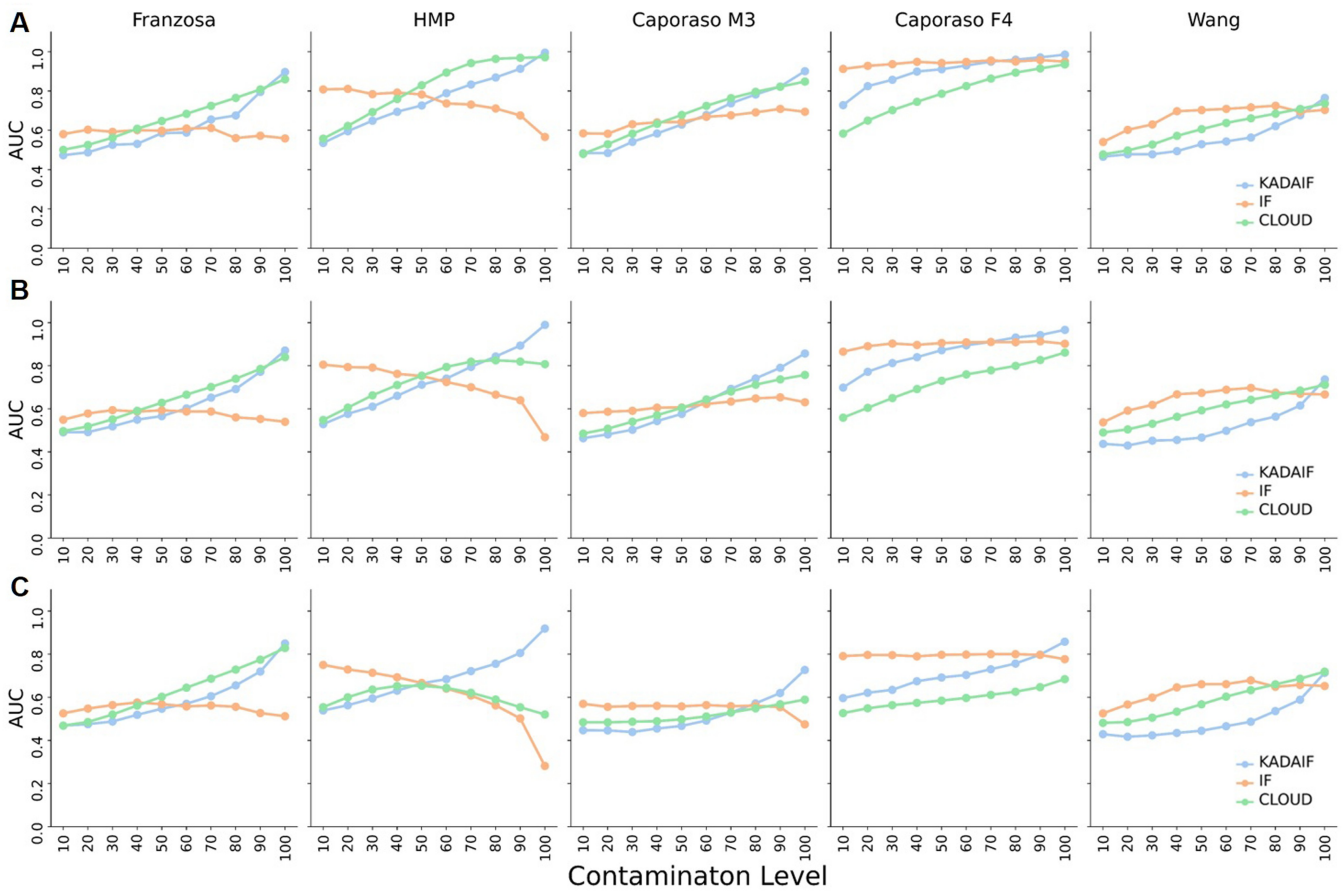
Contamination test results. Comparison between the performances of KADAIF, IF, and CLOUD across different datasets (columns) and prevalence of anomalous samples in the data (A: 4%, B: 10%, C: 30%). *X*-axis in each subplot is the contamination level, p, of each contaminated sample and the *y*-axis represents the AUC obtained when using the anomaly scores for detecting anomalies.

We next examined KADAIF’s ability to identify contaminated samples amongst a pool of “pure” samples. To simulate contamination scenarios, we again generated from each of the datasets above a pool of 50 normal samples, of which a few samples were contaminated by “mixing” the original taxonomic profile of the sample with the taxonomic profile of some anomalous sample. Specifically, in these contaminated samples, the abundance of each taxa was calculated as 1-p of its abundance in the original (normal) sample and p of its abundance in a random anomoulous sample (where p is defined as the contamination level). As above, this process was repeated 50 times, and the performance of each algorithm in detecting the contaminated samples was measured by the AUC. Our results show a consistent pattern: in instances of low contamination levels (ranging from 0.1 to 0.4), wherein the taxonomic profile of the normal sample still comprises the majority of the normal sample, the IF method outperforms KADAIF and CLOUD (Fig. 3). However, in contrast to IF that shows relatively constant (and relatively poor) performances all across contamination levels, KADAIF's and CLOUD’s performances generally improve when the contamination level increases, ultimately surpassing those obtained by IF. This observation makes sense; when the contamination level is low, IF may focus on individual features that were totally absent in the normal profile and present in the contaminating profile, allowing it to effectively identify contaminated samples, whereas KADAIF (similarly to CLOUD) considers community-wide shifts and may thus struggle to detect such samples until contaminating taxa are at sufficiently high abundance. Although the presence of new taxa in contaminated samples may not always apply to real-world contamination, the relationship between the number of taxa in each node and the contamination level that can be detected could be useful for fine-tuning anomaly detection methods when the expected level of contamination is known. It is also important to note that while both KADAIF’s and CLOUD’s performances improve with the contamination level, this improvement is more consistent and more prominent in KADAIF, especially when the proportion of anomalous samples increases. Moreover, in some cases CLOUD tends to exhibit slightly better performances, especially when the proportion of anomalous samples is low, likely due to its nearest neighbors-based approach, which is well-suited for detecting anomalies when they are sparse and markedly distant from normal samples. However, as contaminated samples become more prevalent and subtle community-wide changes emerge, this advantage diminishes and KADAIF’s strategy becomes more effective at capturing broader patterns, often surpassing CLOUD’s performance.

To model a different plausible contamination scenario, particularly one involving contamination by only very few bacterial taxa in the sample that increase in abundance and become dominant, we implemented an additional contamination simulation. Specifically, we again randomly selected 50 samples from the normal group and, for a subset of these, randomly chose 1% of taxa (with a minimum of five taxa) and artificially inflated their abundance, multiplying it by a varying contamination factor. We then applied KADAIF, CLOUD, and IF to this dataset, as before, to evaluate the ability of each method to detect theses contaminated samples. As shown in Note 3, available as supplementary data at *Bioinformatics* online, in this contamination scenario, both CLOUD and KADAIF again outperform IF in most settings. Moreover, in several datasets, and specifically, when the number of anomalous samples is not very low, KADAIF further outperforms CLOUD, with this advantage becoming more pronounced as the contamination factor increases.

### 3.3 Applications of KADAIF to other types of biological data

Since many other types of biological data, such as other omics or single-cell data, share key characteristics with microbiome data, including most notably high dimensionality and sparsity, we next sought to assess whether KADAIF could be similarly well-suited for detecting anomalies in such datasets. We specifically tested KADAIF's performance on metabolomics, methylation, and expression data, using data from three studies to simulate mislabeling scenarios (as described above). In the first two datasets, namely Franzosa *et al.* and Wang *et al.* (which were also analysed above), we used available metabolomics data, with normal and anomalous samples defined as we did in our microbiome analysis. In the third dataset, the TCGA breast cancer dataset (Koboldt *et al.* 2012), we used available methylation and mRNA sequencing data, and defined stage 1 breast cancer tumor samples as normal (139 samples) and stage 4 samples as anomalous (17 samples). Notably, in these analyses, we replaced our use of PCoA with the more generalized PCA, following data normalization and mean centralization.

**Figure 4.**
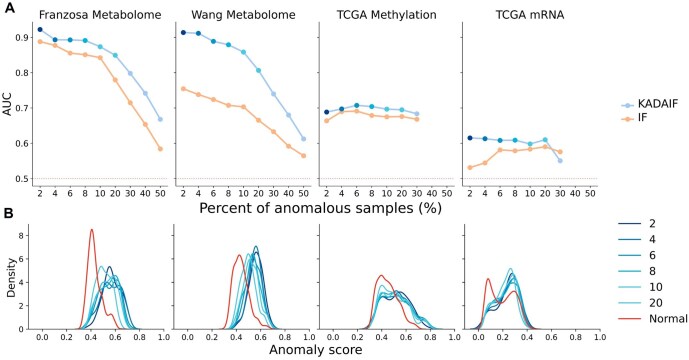
KADAIF successfully detects anomalies in metabolome data. (A) Comparison between the performance of KADAIF and IF in detecting mislabeled samples at various anomaly percentages in metabolome, methylation, and mRNA data. (B) Distribution of anomaly scores for anomalies and normal samples produced based on a mislabeling simulation with 2% of the samples as anomalies.

As shown in Fig. 4A and B, KADAIF tends to assign higher anomaly scores to anomalous samples than to normal samples, and successfully detects anomalies across these data types. Moreover, its performances in this task (as reflected by the AUC) were consistently higher than standard IF across various anomaly proportions, suggesting that KADAIF is better suited for biological data with similar characteristics to microbiome data. Interestingly, we again found that KADAIF is able to identify anomalies in some datasets with considerable accuracy (e.g. AUC of 0.67 in Franzosa *et al.*) even when anomalies comprised 50% of the dataset. This suggests that healthy samples are more difficult to isolate in comparison to, for example, samples from individuals with IBD or ESRD, likely due to the Anna Karenina principle discussed above.

### 3.4 Application of KADAIF to detect disturbance in longitudinal microbiome data

Since severe exogenous perturbations may lead to significant changes in the composition of the microbiome, resulting in an anomalous like behavior, we sought to evaluate KADAIF’s ability to detect such disturbances within longitudinal data. For this purpose, we utilized the longitudinal data from David *et al.* (2014), which extensively followed two individuals sampled on a nearly daily basis over a long period of time. Specifically, subject A in this study was sampled for almost a year, with a substantial portion of this time spent living abroad for approximately 2 months. Moreover, during this trip, subject A experienced two episodes of diarrhea. A disturbance analysis conducted in the original study found that the alterations in dietary patterns (as well as changes in the storage protocol) during the trip, coupled with the occurrences of diarrhea, led to significant variations in the composition of the microbiota.

**Figure 5.**
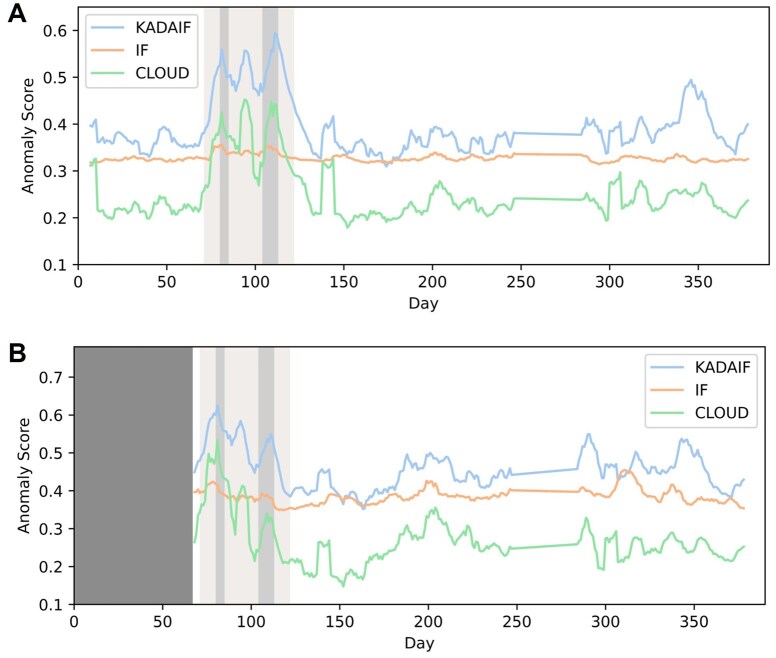
Anomaly scores over time for subject A using KADAIF, CLOUD, and IF. The anomaly score given by KADAIF, CLOUD, and IF to samples from subject A in David *et al.* CLOUD’s scores were normalized to have a maximum of 1. At each time point, the rolling average of the last seven samples is shown to reduce noise and more clearly highlight temporal patterns. Panel A shows anomaly scores for all samples while panel B applies a sliding window framework, considering only the preceding 60 days for each sample. The light shaded gray patch indicates the trip abroad and the green patches represent the diarrhea episodes. The dark grey patch in panel B represents the first 60 days, during which there were not enough preceding samples to compute anomaly scores using the sliding window approach.

Our analysis thus aimed to determine whether KADAIF could effectively identify shifts in microbiome composition during an overseas trip and episodes of diarrhea. To evaluate this, we first applied KADAIF, CLOUD, and IF to all fecal samples from subject A, obtained from the Qiita database (Gonzalez *et al.* 2018), and recorded the anomaly scores assigned to each sample. Our results (Fig. 5A) indicate that all methods assign higher anomaly scores to samples collected during the trip, with peaks during the diarrhea episodes. However, while the anomaly scores from all methods were significantly correlated (Pearson correlation ranging from 0.655 to 0.907, *P*-value <$\;10^{-41}$), KADAIF and CLOUD showed a substantially more prominent increase in anomaly score during this trip and much higher picks in the diarrhea episodes compared to the anomaly scores of other samples. In contrast, IF displayed only a slight increase in the anomaly score relative to other samples, suggesting that it is less effective in detecting such temporary dysbiosis.

To further assess KADAIF’s ability to detect dysbiosis in real time and to demonstrate its clinical relevance for identifying when an individual may require further evaluation, we conducted a similar analysis, but this time using a sliding window framework. Specifically, at each iteration, we evaluated one focal sample but used, as our dataset, only the samples collected within the 60 days preceding that sample (instead of analysing all samples). We then applied KADAIF, CLOUD, and IF to each such sample group, examining the anomaly score assigned to the focal sample as a measure of recent changes in microbiome composition. This approach can be conceived as a real-time detection scenario of microbiome dysbiosis, when at each time point, only recent samples are considered. As shown in Fig. 5B, KADAIF’s anomaly score still peaked during both diarrhea episodes, while this pattern almost vanished completely with standard IF. CLOUD’s results were still correlated with those obtained by KADAIF (Pearson correlation =0.77), but its ability to accurately capture the second diarrhea episode markedly declined in comparison to KADAIF. This result underscores KADAIF’s capacity for real-time dysbiosis detection, highlighting its potential for clinical applications.

### 3.5 Application of KADAIF to partition case versus control samples with the Anna Karenina principle

In recent years, multiple studies have observed the occurrence of the Anna Karenina principle, inspired by the famous opening line of Leo Tolstoy’s novel, in various disease-associated microbiome shifts (Zaneveld *et al.* 2017, Ma 2020). Simply put, this principle suggests that microbiome compositions are more consistent among healthy individuals than among individuals with a certain disease, with the assumption that imbalances in a given disease can arise through multiple distinct pathways, leading in turn to greater variability.

Indeed, as noted above, when KADAIF was applied to case-control datasets, it successfully separated case samples from control samples, even when the number of case samples and control samples was similar. This suggests that KADAIF’s approach to anomaly detection is also sensitive to such disease-associated variability and can use this elevated variation to distinguish disease samples from normal samples. To gain a deeper understanding of this behavior, we used data from the MetaCardis cohort (Fromentin *et al.* 2022), which includes gut microbiome samples from individuals with various cardiometabolic conditions of varying severities, alongside healthy individuals. This diverse dataset allows us to assess KADAIF's ability to distinguish different conditions and severities based on this within-group variability alone.

**Figure 6.**
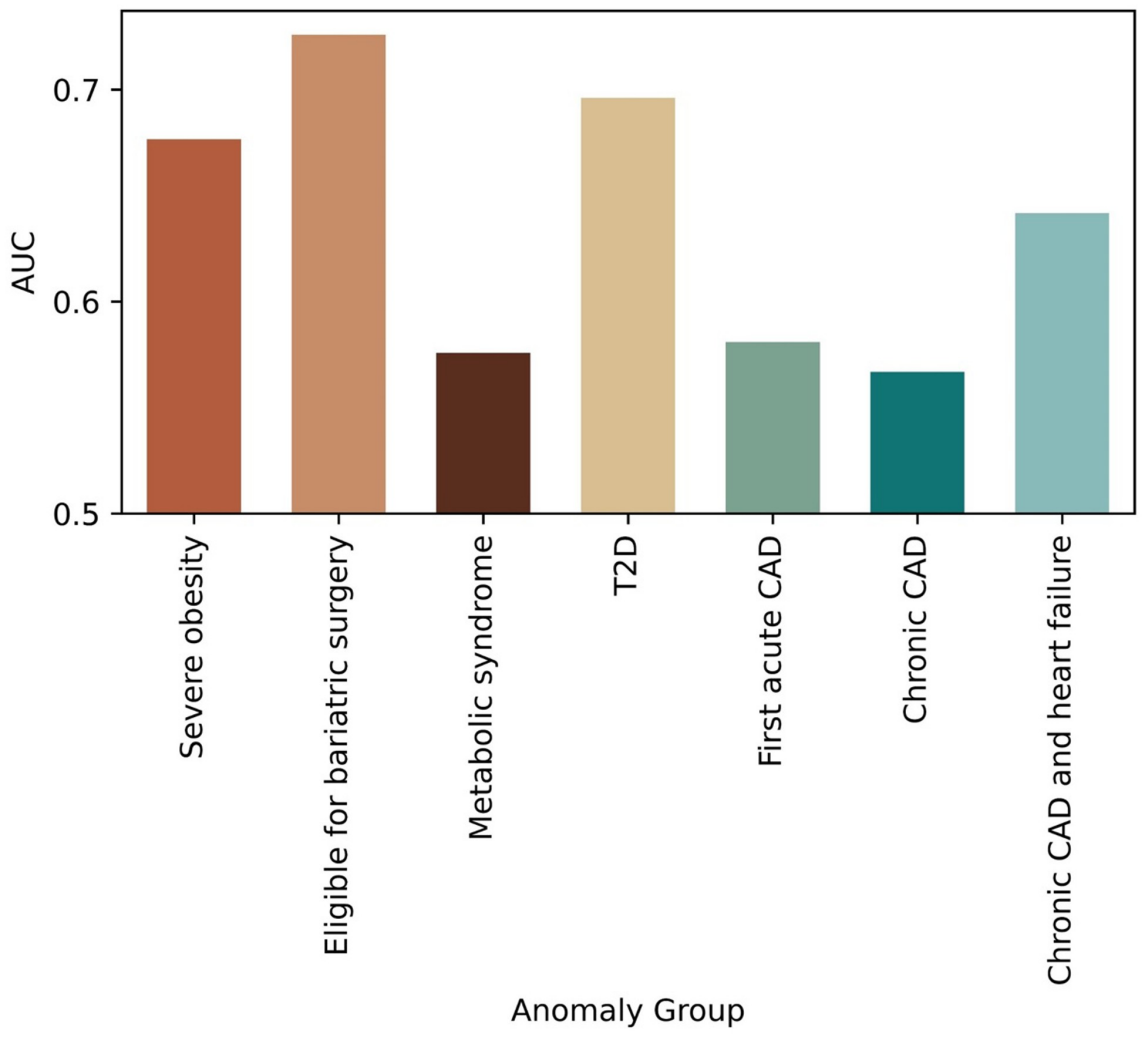
AUC of partitioning case versus control samples based on KADAIF’s anomaly scores, for different health conditions.

We first used these data to generate multiple case-control datasets, each containing the healthy samples from this cohort and an equal number of samples from one of the medical conditions included. We then ran KADAIF to obtain anomaly score for each sample and used these scores to try and partition case samples from control samples. As shown in Fig. 6, anomaly scores assigned to samples from healthy individuals are indeed lower compared to those assigned to samples from any other condition, resulting with an average AUC for classifying cases versus control across conditions of 0.638. This analysis confirms that KADAIF effectively captures the greater variability that is present amongst individuals with a given condition compared to the more uniform healthy state.

Moreover, while all conditions could be identified to some extent (AUC >0.5), we found a clear relationship between the ability to partition case versus control samples and the severity of the condition. Specifically, obese individuals eligible for bariatric surgery were identified more accurately than those not eligible (AUC of 0.726 and 0.677, respectively), individuals with type 2 diabetes were identified more accurately than those with metabolic syndrome (Meamar *et al.* 2020) (AUC of 0.696 and 0.576, respectively), and individuals with chronic CAD with a history of heart failure identified more accurately than those with first acute CAD and chronic CAD without prior heart attack (0.642, 0.58, and 0.567, respectively). Moreover, applying KADAIF to datasets that include equal number of samples from two or three conditions alone (without controls), further showed that KADAIF can in fact partition obese individuals eligible for bariatric surgery from obese individuals (AUC 0.581), type 2 diabetes individuals from metabolic syndrome individuals (AUC 0.639), and individuals with chronic CAD with heart failure from patients with chronic CAD without heart failure (AUC 0.561) and from patients with first acute CAD (AUC 0.565).

## 4 Discussion

In this study, we introduced a novel anomaly detection algorithm, KADAIF, that generalizes the IF algorithm and is specifically designed to cope with various characteristics of microbiome data, including sparsity, high dimensionality, and complex interactions. Specifically, KADAIF selects multiple features and projects them on a principal component to split samples in each node during the tree construction process, instead of relying on a single feature as in the standard IF algorithm. This extension allows KADAIF to better account for sparsity and interdependencies in the data.

We compared KADAIF’s performance with that of IF and CLOUD, one of the few anomaly detection tools developed specifically for microbiome data, by simulating two common scenarios that can distort true biological signal: mislabeling and contamination. In the mislabeling scenario, KADAIF clearly outperformed both IF and CLOUD across various datasets and percentages of anomalous samples. In the contamination scenario, the differences between the methods were more nuanced, with IF outperforming KADAIF and CLOUD at low contamination levels. However, as contamination level increased, KADAIF consistently improved in its ability to detect contaminated samples, while IF did not show much improvement. We thus argue that KADAIF’s approach may still be more favorable, especially when the level of contamination is variable or unknown. We also note that CLOUD's behavior in this simulated contamination scenario was generally similar to KADAIF's, although less consistent, with smaller and more noisy improvement in performance when the proportion of anomalous samples increased.

Beyond these controlled scenarios, we explored additional applications of KADAIF to microbiome and other biological data. First, we showed that KADAIF is successful in detecting anomalies in a variety of biological datasets, especially those that share similar characteristics to microbiome data. It should also be noted that during the analyses on these non-microbiome datasets, we used PCA instead of PCoA. This use of PCA in fact supports projecting new samples (i.e. samples that were not included in the original analysis) onto the same latent space and using the constructed forest, enabling the calculation of anomaly scores for previously unseen samples. This capability, in turn, expands KADAIF’s possible applications, including for example, using KADAIF for novelty detection, determining whether new samples exhibit patterns not previously observed in the data (Chandola *et al.* 2009, Su *et al.* 2018). We also tested whether KADAIF could detect disturbance in longitudinal microbiome data, demonstrating that KADAIF outperforms IF in identifying episodes of diarrhea in daily-obtained microbiome data. Lastly, we utilized the occurrence of the Anna Karenina principle in microbiome data, showing that KADAIF can partition case versus control samples based on the increased variability in disease.

Importantly, despite the above favorable findings, we note that anomalous behavior may not be universally defined and may stem from a large variety of factors. For instance, anomalies may stem from batch effects, technical errors, or processing biases. Our study focused only on specific (and common) types of anomalous samples, namely mislabeled and contaminated samples, and future research may be required to explore alternative definitions of microbiome anomalies and to investigate how such anomalies are best detected.

Overall, however, we believe our findings provide strong evidence that KADAIF effectively detects anomalies in microbiome and other omics datasets, making it particularly well-suited for high-dimensional, sparse biological data. More importantly, we hope that introducing KADAIF will encourage broader adoption of anomaly detection in microbiome research, as is common in other computational domains. Incorporating this step during preprocessing can enhance data quality, reveal more precise biological signals in downstream analyses, and ultimately improve the reliability of biological inferences.

## Supplementary Material

btaf520_Supplementary_Data

## Data Availability

An implementation of KADAIF, as well as all the code used for the analysis, is available on GitHub (https://github.com/borenstein-lab/KADAIF).
