# Supplementary material for: KADAIF: an anomaly detection method for complex microbiome data
Source: Bioinformatics. 2025 Sep 19;41(10):btaf520. doi: 10.1093/bioinformatics/btaf520 (PMC12757010; doi:10.1093/bioinformatics/btaf520)
Supplement: btaf520_Supplementary_Data [file btaf520_supplementary_data.pdf]

Supplementary Note 1: Hyperparameters and configuration tuning

To fine-tune KADAIF's performances, we tested various parameters and configurations to optimize KADAIF for further analysis. These parameters pertain mostly to the feature selection process, including sampling with or without replacement, the number of selected features, and whether features are selected uniformly or proportionally to their relative abundance. We also tested parameters concerning the dimensionality reduction process, specifically whether the algorithm should use only the first principal component or additional components. KADAIF's anomaly detection performance across these configurations was evaluated using the mislabeling simulation described in the main text, with anomaly percentages ranging from 2% to 10%. The tuning process was conducted using the four datasets from the mislabeling test described in the main text.

We first evaluated whether features should be drawn uniformly in each node or based on their average relative abundance in the dataset. As shown in Figure S1A, uniform feature selection consistently outperforms selection based on mean relative abundance in all datasets except for in HMP where the two approaches have similar performance. Therefore, we chose to draw features uniformly. We also examined whether there is a difference between selecting features with vs. without replacement. As shown in Figure S1B, our results did not reveal a clear advantage to either approach, and we thus opted to proceed with feature sampling with replacement.

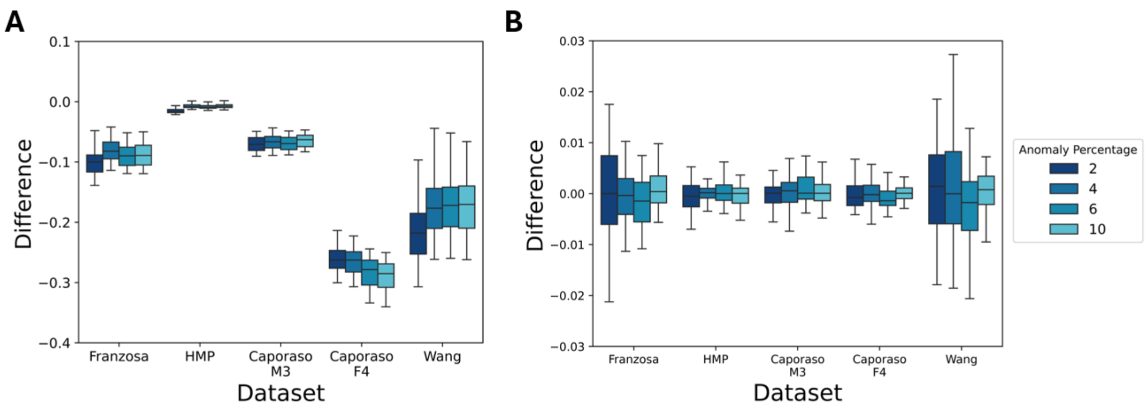

Figure S1: Difference in AUC for the mislabeling test between two otherwise identical experiments, differing only in (**A**) the method for selecting features (AUC when selecting features with probability proportional to their average relative abundance, minus the AUC when selecting with uniform distribution); and (**B**) selection with replacement minus selection without replacement. AUC values are shown across various datasets and anomaly percentages.

Next, we examined how the procedure for selecting principal components (PCs) after dimensionality reduction (with PCoA) affects KADAIF's performances. We specifically evaluated three options: the first considers only the first PC, the second selects one of the first 20 PCs uniformly at random, and the third again selects a random PC from the first 20 PCs but with probability proportional to the proportion of explained variance. As evident from Figure S2, there was no clear difference between the three methods, and none of the observed differences was statistically significant. However, we opted for the last option described above, which prioritizes PCs based on explained variance. Despite being slightly more time-consuming, this approach balances the advantages of the other two: it allows selection of one of several available PCs, potentially capturing patterns beyond the first PC, while still giving more weight to the first PC, which retains the most information.

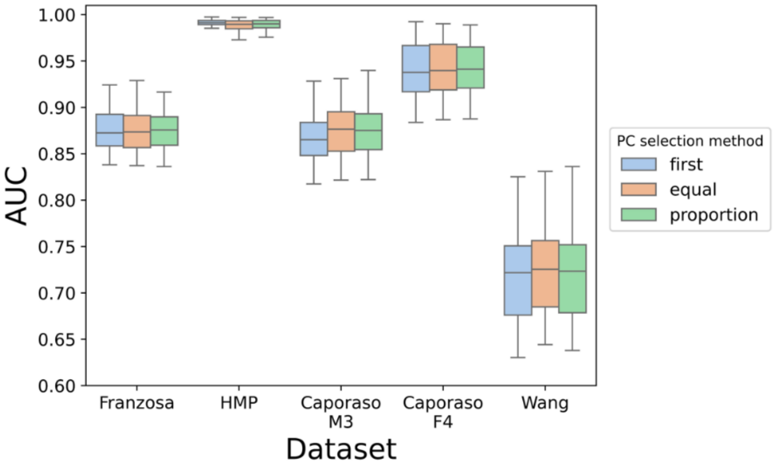

Figure S2: AUC performance of KADAIF across the four datasets using three methods for selecting the principal component (PC) to be used.

We set out to determine the optimal number of features to be considered at each split. As demonstrated in Figure S3, all datasets exhibit a similar trend, though with varying magnitudes: Specifically, when the number of features is still low, KADAIF's performance improves as more features are added. However, beyond a certain point, this trend is being reversed, and the AUC declines with the addition of more features. This initial improvement is less evident in the Caporaso *et al.* dataset, which contains only 335 features, limiting our ability to test very low feature counts. Based on these findings, we chose to select ~1% of the features per split (with a minimum of 5), as such a set provides a sufficient number of features for generalization, while avoiding performance degradation. We also tested a configuration where the number of selected features was randomly drawn from a uniform distribution between one and the total number of features in the dataset, but this approach performed worse than the ~1% configuration.

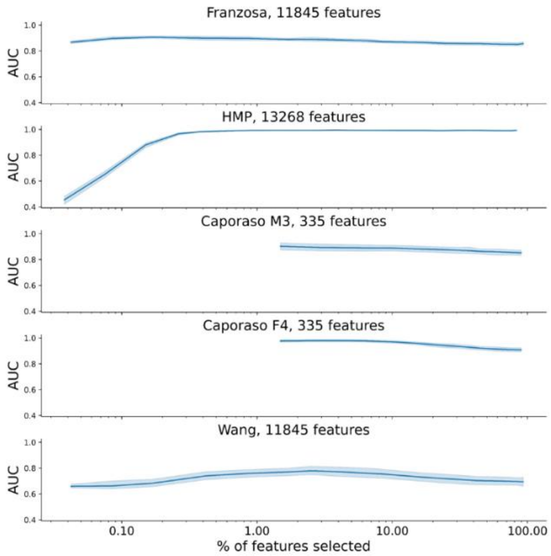

Figure S3: Performance of KADAIF across different numbers of selected features per split. Each panel represents a different dataset, showing the average AUC across all anomaly percentages as a function of the percentage of features used at each split

Finally, we confirmed that the anomaly scores assigned by KADAIF to samples labeled as normal do not change substantially with the percentage of anomalous samples in the dataset. Indeed, examining the distribution of these anomaly scores across different datasets and anomaly percentages suggests that these scores are highly stable (Figure S4).

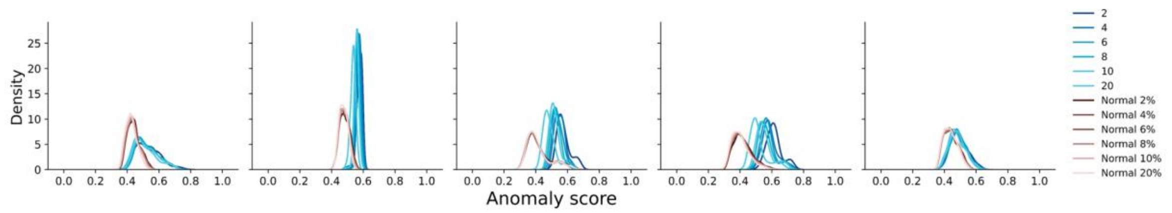

Figure S4: Distributions of KADAIF's anomaly scores for samples labeled as normal and anomalous across datasets and anomaly percentages, including the score distribution of the normal samples across various anomaly percentages.

Supplementary Note 2: Evaluating different dimensionality reduction methods and distance metrics

As noted in the description of KADAIF, one of the algorithm's steps involves applying dimensionality reduction to the selected subset of features. In our analyses, we specifically used Principal Coordinates Analysis (PCoA) based on Bray-Curtis distances, which is a common practice in microbiome data analysis.

Here, we set out to further evaluate the effect of using different distance metrics or different dimensionality reduction strategies on KADAIF's performance. To this end, we conducted an additional set of simulations, using again the mislabeling simulation described in the main text, but this time compared the default configuration of KADAIF (relying on PCoA and Bray-Curtis distances) to two alternatives: The first uses PCoA based on weighted UniFrac distances (which accounts for phylogenetic relationships between taxa) instead of Bray-Curtis, and the second uses standard Principal Component Analysis (PCA) based on Euclidean distances. Since UniFrac requires a phylogenetic tree, we restricted this alternative to the two shotgun metagenomics datasets analyzed in our study (Franzosa *et al*. and Wang *et al*.), for which high-quality trees were already constructed as part of our data processing pipeline.

As shown in Figure S5 below, across all datasets and almost all tested fractions of anomalies in the dataset, using PCoA with Bray-Curtis consistently yielded higher AUC values than those obtained using either PCoA with UniFrac or PCA with Euclidian distances. It should also be noted that UniFrac computation was substantially slower (up to two orders of magnitude) than Bray-Curtis in our implementation, markedly increasing runtime and limiting scalability. Based on these observations, and considering the strong performances, low computational cost, and broad applicability obtained using the Bray-Curtis metric, we retained it as the default option in KADAIF.

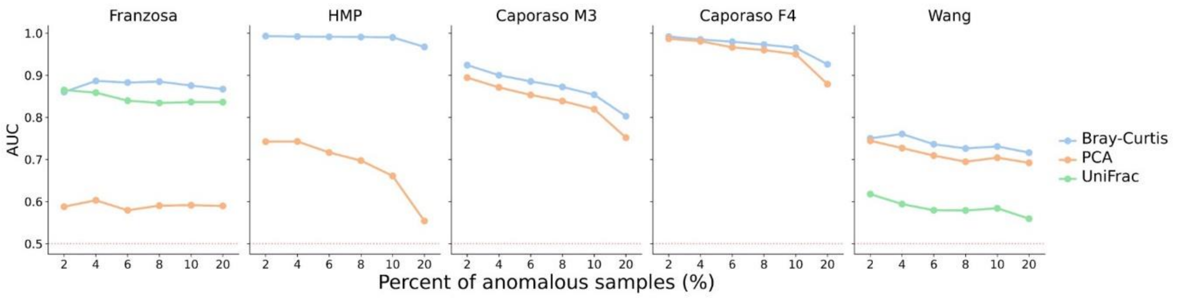

Figure S5: Performance of KADAIF using various dimensionality reduction approaches and across different anomaly levels. Results are shown using PCoA with Bray-Curtis distances (KADAIF's default), PCoA with weighted UniFrac distances (applied only to the Franzosa *et al*. and Wang *et al*. datasets), and PCA with Euclidean distances.

Supplementary Note 3: Simulating contamination by a small number of taxa

We have designed and implemented an additional contamination simulation to model a different form of realistic contamination scenarios, particularly those where only very few bacterial taxa contaminate the sample and become dominant. For this simulation, we first randomly selected 50 samples from the normal group as before. Then, for a subset of these samples, we randomly chose 1% of taxa (with a minimum of 5 taxa) and artificially increased their abundances by a varying scaling factor to mimic a contamination event dominated by a small number of microbial species. To exclude low-prevalence taxa unlikely to be influenced by this factor, we filtered the data to retain only taxa present in at least 80% of samples in the shotgun datasets and at least 20% in the 16S datasets before this scaling process. We finally, as before, evaluated the ability of KADAIF, CLOUD, and IF to identify these contaminated samples based on the AUC obtained from 50 repeated simulations. This new simulation framework represents a plausible, and potentially more biologically relevant contamination scenario. As shown in Figure S6, both KADAIF and CLOUD again outperform IF in most settings. However, in these simulations, KADAIF often outperforms CLOUD, especially when the number of anomalous samples is not very low (e.g., Figure S6B-C), and this advantage further increases as the contamination factor increases.

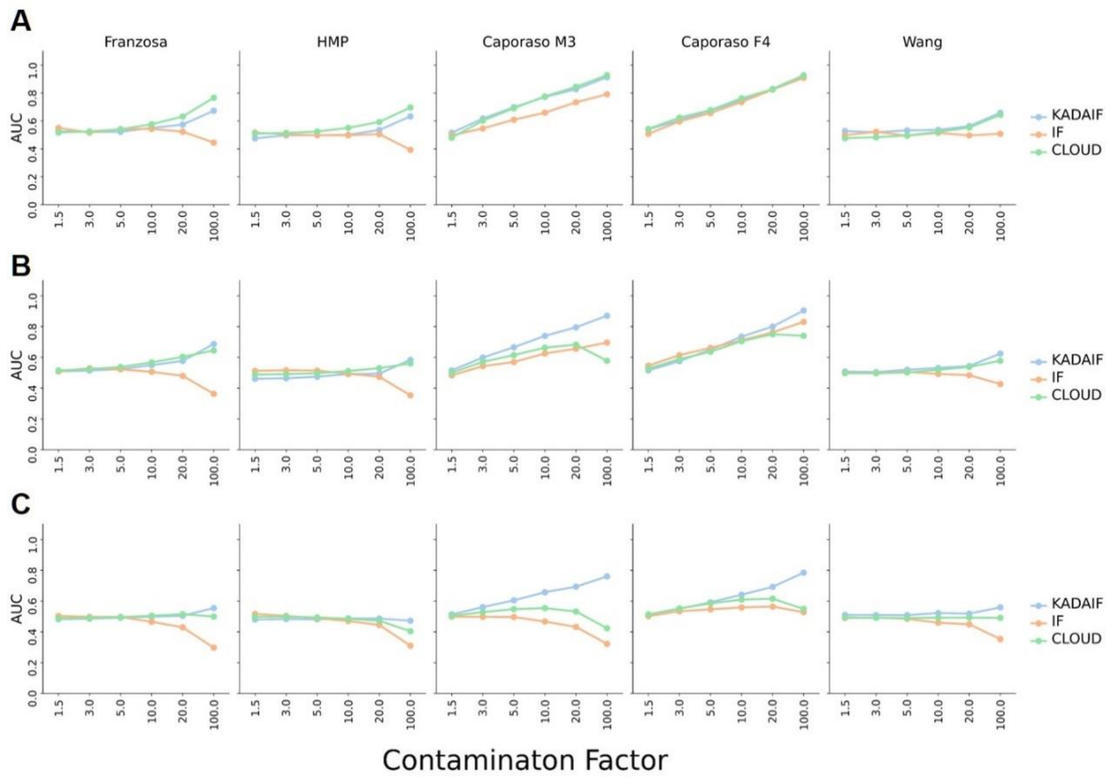

Figure S6: Contamination simulation based on a small number of taxa. Comparison between the performances of KADAIF, CLOUD, and IF across different datasets (columns) and prevalence of anomalous samples in the data (**A**: 4%, **B**: 10%, **C**: 30%). The x-axis in each subplot is the contamination factor by which the few contaminating taxa are multiplied and the y-axis represents the AUC obtained when using the anomaly scores for detecting anomalies across 50 simulations.
